# How to deal with the past? How collective and historical trauma psychologically reverberates in Eastern Europe

**DOI:** 10.3389/fpsyt.2023.1228785

**Published:** 2023-08-24

**Authors:** Andreas Maercker

**Affiliations:** Department of Psychology, University of Zurich, Zürich, Switzerland

**Keywords:** historical trauma, traumatic stress, distrust, commemorating, trauma healing, story telling

## Abstract

Traumatic stress studies have recently addressed the issue of ‘historical trauma’ that well explain the impact of collective or totalitarian trauma. The example of former communist Eastern Europe shows that there are many individual and socio-psychological consequences that still have effects today. This paper summarizes concepts and findings on ‘historical traumas’ that describe such long-lasting effects. The focus is on the side of the victims and their family descendants and thus also on the moral heirs of the dissidents, e.g., the Russian NGO Memorial. Analogous to developments in psychotraumatology, increasing knowledge in this area can explain psychosocial pathologies but also help develop effective remedies. This includes the development of a culture of remembrance, socio-therapeutic interventions and increased sensitivity towards those patients and clients who have such a personal legacy.

## Introduction

The former communist countries of Eastern Europe are currently facing multiple layers of traumatic experiences and challenging transitions, the most recent being the Russian war of aggression on Ukraine. This conflict rightly receives highest media attention and is the focus of initial psychosocial research to understand the extent of the suffering involved. However, this paper deliberately addresses an earlier layer of historical trauma in this region, namely the totalitarian trauma of Soviet communism. It essentially consisted of the Stalinist and post-Stalinist repression that lasted for almost 70 years in the Soviet Union and for over 45 years in the regions of Eastern Europe it had subjugated in the 20th century. The current paper aims to present a range of mental distress related to this past, focusing on the survivors, victims, and their descendants. It draws on a wide variety of sources, all of which provide evidence that can be used in traumatic stress research, psychotraumatology, psychosocial treatments, and other forms of healing. Those interventions that promise to be effective in terms of the concept of historical trauma, however, still come from other contexts and regions of the world where they were developed to overcome the consequences of genocide and ethnic oppression.

Subsequent to introductory case studies, this article presents a definition and an overview of the concept of historical trauma (abbreviated as HT), along with presentations of the psychosocial consequences and an outlook on potential remedies, such as sociotherapies and a culture of remembrance.

## Clinical cases and psychosocial pathologies

The trauma sequelae commonly reported by survivors of atrocities of the Stalinist repressions can be subsumed as man-made traumatic experience by the diagnoses of post-traumatic stress disorder (PTSD) or complex PTSD. However, their subjective suffering goes beyond these symptom patterns as it is inextricably linked to certain historical constellations. The following case studies show this and also indicate that trauma consequences also affect the societal level. Thus, the historical context in which individual traumas occur can often provide a better understanding. The examples are intended to be prototypical of those persecuted under Stalinism and post-Stalinism and are limited to a diagnostic-conceptual classification, as it stands at the beginning of every medico-therapeutic case conception.

An 85-year-old woman living in the republic of Georgia reports a happy childhood. However, when she was a teenager, persecution measures began against her parents and older brothers. She witnessed their multiple imprisonments and then had to take care of the younger children. Her father and brother disappeared completely, and she believed that they had been killed. She and her siblings were publicly labeled as “enemies of the people.” She was forbidden by the authorities to talk about her experiences, which she kept to herself for 50 years. Since then, she has strong fears, is easily frightened, and has repeating nightmares. Even nowadays, she is afraid when a car stops in front of her window; then, she can no longer fall asleep. Because of her many fears, she had lived a very secluded life and also brought up her children that way. She continues to feel the effects of the fear she experienced for her family members’ lives, and the resulting damage and grief have not dissipated (1).

Her report reveals that she is experiencing a range of PTSD symptoms together with ongoing grief and external pressure to remain silent about her experiences for fear of being sanctioned. The restricted quality of life that still exists today can be recognized. The reports of other concerned persons contain disturbances that can be diagnosed according to the new International Classification of Disorders, version 11 (ICD-11) of the World Health Organization’s as complex PTSD (2).

The 45-year-old patient, who was imprisoned for political reasons in the former GDR at the age of 21 and spent 8 years in prison, reports in his own words: *“I am no longer like that, I have become different […]. Many things simply do not interest me anymore. But when something comes up, I overreact […]. Either you freeze in front of it and do not dare to move, or you revolt against it and do not take it seriously at all. The middle ground, what would be appropriate, is missing from me […]. And when I see again that the [former perpetrators] have it good, that causes me such a massive stomachache […] As long as I live, I will hate everything that has to do with them […]. I sometimes got very angry with my wife for not understanding… very, very angry… You have that victim mentality, you just expected your environment to understand you. But that is not true, and there was such a wall of silence, a complicity of silence. One continued to curl up into a ball…”* (2).

As compared to the first case report, additional areas of psychological functioning have been affected. Moreover, in this instance, the former political dissident draws connections between his experiences in prison and trauma-related dysfunctions that persisted long-term even after the end of the communist rule. A knowledge of historical contexts would help his social partners in everyday life to adequately understand his symptoms.

Another level of diagnostic-conceptual classification can make use of the concepts of ‘totalitarian trauma’ existing in the literature (1) or the ‘post-communist syndrome’. The latter (consisting of ‘learned helplessness’, ‘incivility’, and ‘lack of civic virtues’) is aimed to describe the overall situation of the directly persecuted and the general population in the countries within the post-communist transition (3). The ‘post-communist syndrome’ describes changes that tend to persist over a long period of time and that may occur to an entire community:

“Anxiety, caused by repeated purges by the secret police and a fear of being reported for some real or perceived misdeed, resulted in distrust and in widespread mutual suspicion. In combination with frustration arising from a scarcity of consumer goods, this suspicion further contributed to a general decline in civility […]. Official repression of formerly functional moral systems such as religion, local community networks and voluntary associations led to a further decline in morality […]. Disillusionment with politics led to pessimism and cynicism and to a lack of hope and of vision for the future.” (3).

All the conditions described here lead to a special need for medical interviewing and psychosocial intervention, as it is essential to understand both the HT contexts and the psychopathology. A historically ill-informed treatment procedure can lead to feelings of misunderstanding and resentment. It could already be observed in the last decades that Eastern European patients - and on another level also the clinicians from these countries - felt misunderstood when ill-informed mental health providers for example offered trauma treatments, without understanding the unique mental health issues that are shaped by Eastern Europeans’ specific historical experiences (4, 5). The author’s own clinical observations, including those from supervision and the observations of colleagues from the former Soviet regions of influence in Europe, provide multiple support of this. The following definitions and research results may serve as a basis for historical-trauma-informed treatment, whether at the individual, family or community level.

## The historical trauma concept

The concept of historical trauma (HT) was coined around the year 2000 after a connection between past collective traumatization and certain forms of suffering were identified in other parts of the world. The social workers and scholars Brave Heart and DeBruyn initially linked the concept to the consequences of colonization on American Indians but also made reference to the effects of the Holocaust and the according research (6).

HT can be defined as follows:

Prerequisites:

- Collective trauma, e.g., enslavement, severe oppression, wars.
- Persistent discrimination, racism, or marginalization.

Consequences:

- Intergenerational trauma effects and individual psychopathologies.
- Today’s social disadvantages and inequalities.
- Social pathologies such as high mistrust, bitterness, etc.
- No or at most a dysfunctional collective victimhood narrative.

Many authors have since provided empirical evidence for the various facets of the concept. To this day, American Indigenous mourn various cultural losses that date back to both before and after the final combats against their ethnic groups around 1890 in the United States. One sad example is the establishment of the residential school system for their children by 1970, which separated them from their parents and was aimed to deprived them of their identity (7). here is substantial evidence of individual and structural discrimination against these ethnic groups, which has been linked to an increased prevalence of psychopathology (8, 9). As a result, the affected groups exhibit more pronounced current psychopathologies than the surrounding majority society (7, 10). For intergenerational trauma effects, there are findings in the context of the Holocaust. Here, social pathways of transmission, such as communicative and parenting disturbances, have been highlighted in particular (11, 12). Studies have shown that contemporary social pathologies are characterized by general mistrust, loss of identity and values, and perceived institutional betrayal (13, 14). For a significant period of time, adequate recognition of the historical trauma experienced by American Indigenous people had been absent, thus impeding the initiation of the healing process (15, 16).

Even though the concept of HT originated outside Europe, it can also be used to better understand the psychological development and disorders in victims of Stalinist and post-Stalinist repression.

## Totalitarian trauma in Eastern Europe

As for some historical facts of Stalinist and post-Stalinist repression in Eastern (and parts of Central) Europe: The onset of systematic terror in the Soviet Union can be traced back to the accession of Josef Stalin. Until Stalin’s death in 1953, 8.2 million people were directly killed, 7.5 million people were deported, and 5 million were put in prisons. Approximately one in eight adults is estimated to have been a victim of some form of repression, meaning that there were victims in every other family during this phase (17). In the post-Stalin phase after 1953 until the end of the Soviet Union, coercion and political imprisonment continued, although the numbers dropped to the still alarming number of tens of thousands of victims (18). Today, the descendants of these people live in 15 independent countries of the former Soviet Union. In Ukraine, the Stalin era brought another massive deportation: the human-caused famine of the Holodomor of 1932 and 1933, which killed 4 million people, a tenth of the population (19). In Lithuania, which had been part of the Soviet Union since 1940, the deportations were exceptionally high – 10% of the population.

In the Soviet sphere of power in Eastern and Central Europe, the Stalinist and post-Stalinist eras were also devastating. In Poland, up to 150,000 people were killed by the Soviets or their own communist government, and 300,000 people were political prisoners (17). In the then East German Democratic Republic (GDR), there were 200,000 political prisoners by 1989, not including other political victims (deportees, victims of the so-called decomposition, and youth in penal camps) (20). Since the Stalinist and post-Stalinist periods of repression occurred almost 100 to 35 years ago, up to four generations have lived since these haunting events.

## Method of reviewing supporting findings

The concept of HT and related conceptions still has very little thematic coherence. Therefore, the search for relevant literature was spread very broadly with the aim to gather and synthesize available literature from different Eastern European countries (East Germany, Georgia, Lithuania, Poland, Romania, and Ukraine) and various empirical sciences (psychiatry, psychology, social work, social or political science). In form of a scoping review, in the following presentation of results, quantitative and qualitative analyses, as well as big-data analyses are utilized. The sources searched include Medline, Scopus, SocINDEX, PsycINFO, JSTOR and Google Scholar as well as information from personal contact with professional colleagues in Eastern and Central Europe (see acknowledgment section). In the databases, search descriptors included (in fields keywords or title): historical trauma OR totalitarian trauma OR collective trauma OR Stalinist* OR post-Stalinist* OR Holodomor OR repression OR marginalization OR intergenerational OR transgenerational OR trust OR mistrust OR distrust OR embitterment OR survival strategies OR survival messages. Subsequently, this general search was refined by blocking some of these terms together with: (AND) intervention OR treatment OR psychotherapy, as well as (AND) the specific country names of Eastern Europe listed above.

## Methodological limitations, positionality statement

This paper could not be consistently based on current empirical evidence. Where elements of the above HT definition have not been the subject of previous empirical research (quantitative or qualitative), the authors’ own clinical experiences (as described in the introduction) and those of experts/colleagues from the countries concerned have been included to bridge the gaps in empirical research. It is hoped that these gaps will subsequently be filled by new thorough research.

The author’s positionality on the topic is as follows: My biographical involvement in the topic is that I grew up in the GDR, at that time characterized by post-Stalinism. In my own family, along with social network, there were many more victims of this political system than protagonists or supporters of it.

## Relevant HT research findings

Results obtained from the literature review and from colleague consultation could be categorized into five areas based on the theoretical HT criteria mentioned above: (a) collective traumatization, (b) persistent discrimination and marginalization, (c) manifestations in the victim’s generation, (d) intergenerational transmission and survival messages, (e) generalized mistrust and political sentiments. The following is a brief narrative summary of the most illustrative findings. Complementary to the presented findings, a table with the individual studies’ methods and results can be found in the appendix.

### Collective traumatization

The corresponding papers summarized the extent, historical developments and specifics of this repression. The context was described in which the entire society was exposed to massive terror accompanied by indoctrination, brainwashing, and moral confusion about basic human values (1, 21). The political regimes in the concerned countries functioned by “totalism, randomness, and unpredictability of terror…. Anyone can become a victim at any time; no one is safe” (21). Javakhishvili described how sociological role types narrow down to just three during totalitarian regimes: either one is a victim (one is threatened with death or imprisoned for many years), a perpetrator (one supports the terrorist government), or a bystander (one tries to remain on the outside) (1). These three roles can be swapped among each other, as was the case in the Stalinist system, where many perpetrators or bystanders later became victims, and some victims became perpetrators in the subsequent phase of terror. Recently, Javakhshvili and Volkan recently utilized this concept to provide a psycho-historical interpretation of Vladimir Putin’s biography (22, 23). In the following the focus is placed on the victims and their descendants.

### Persistent discrimination and marginalization

HT and its aftereffects only arise under conditions of persistent marginalization and discrimination (because otherwise social healing would occur). There is supporting evidence for this as well in the groups considered here. Literature on victim groups of post-Stalinist violence in Eastern European countries often highlights that the personal rejection of the majority society towards these victims is pervasive and particularly painful (4, 24, 25).

Banning the memorial organization Memorial for the Victims of Stalinism in Russia in the autumn of 2021 was the vicious endpoint of today’s discrimination against victims in Russia (the organization received the Nobel Peace Prize 6 months later for its essential work). Even before this ban, there were political reprisals against the commemoration; moreover, research showed that the general public in Russia increasingly marginalizes the memory of those who were affected by the regime. The still largely independent Levada polling institute (2022) showed that Stalin’s actions (“cruel and inhumane tyrant, guilty of the deaths of millions of innocent people”) were viewed positively by 32% of the population in 2008 and that this approval increased to 56% in 2018. In Russia, the political changes towards today’s authoritarian form of government are thus associated with an increasing appreciation of the main originator of the repressions – it also needs to be emphasized here that since Russia’s increasing aggression since 2014, there has been no research at all on the trauma consequences of Stalinism and post-Stalinism.

With regard to marginalization, a representative household survey in the East German state of Thuringia (former part of GDR) in 2007 found that 33% of respondents were against financial compensation payments for injustices suffered by former political victims (prison sentences, deportations, expropriations) and 19% stated that the whole issue of coming to terms with the past interested them personally only very little or not at all (26).

### Manifestation in the victim generation

Those who experienced repression could be questioned about their psychological states from the end of communism in 1990. A broad range of outcomes was observed, including severe mental impairment, moderate or temporary impairment, and even resilient mental health characterized by post-traumatic growth. In the former communist East Germany, around 60% of individuals who were released from prison developed PTSD immediately after their release, yet this figure decreased to 30% over the course of several decades (27). Among former prisoners, there was a “waxing and waning” of symptom burden and diagnostic state over the course of 15 years, depending on social-interpersonal factors and coping processes (28, 29). For example, perceived recognition as a victim was a predictor of lower symptom burden. Yet, pronounced comorbidities were present, e.g., social phobias, major depression, and substance use disorders (28). Post-traumatic stress disorder symptoms were also found in persecuted groups and their descendants in studies from Eastern Europe (Lithuania, Poland, Romania, Russia) (30–34). Ukrainian psycholinguistic studies of Holodomor victims 70 years later also showed that persistent anxiety was associated with certain narration features (35, 36).

A number of clinical indications emerged that the previously prevailing formulation of PTSD as a memory and fear-related disorder fell short because it did not take into account the persistent personality changes of those affected (37). Accordingly, the diagnosis of complex PTSD, introduced in the ICD-11 of 2019, is better suited to capture the complexity of frequent persecution-related trauma consequences. This would allow for a broader research focus on changes in personality and its functionality, and would also open up a transdiagnostic perspective.

### Intergenerational transmission and survival messages

The two possibilities of intergenerational transmission described in the general trauma literature are the epigenetic and the social pathway. For the topic of HT, there have so far only been studies on the social pathway, which describes the transmission through means of social-interpersonal interaction in various potential tracks.

A communication style that is characterized by concealment or avoidance of talking about the traumatic experiences is a frequent finding. This avoidance can occur in several subsequent generations. An East German study showed that if the 2nd generation tends to keep quiet about Stalinist injustice and mistrust, then the 3rd generation is more likely to do the same (38). In addition, the general family cohesion in these families is poor. A Russian study with adult grandchildren of repression victims showed that two-thirds were completely “cut off” from information about the persecution of their grandparents, even though they were all in close relationships with them (39). Higher levels of family concealment were associated with poorer physical and mental health and higher divorce rates in the grandchild generation.

Qualitative studies exemplarily point to further changes: A 3-generation Ukrainian study found that representatives of the second and third generations said they had learned ‘fear to take action’ from the first generation, expressed in the fear to change the status quo, passivity, and ‘slave mentality’ (30). At the same time, they were taught indifference towards others and social hostility. Similar family instructions for the intergenerational transmission are subsumed as ‘survival messages’. These are narrative instructions on how to conduct one’s life, which are usually geared towards coping with coming new misfortunes according to the motto ‘Learn something from our misfortune’. Javakhishvili lists messages such as “keeping a low profile” in order not to become a victim again in the case of new political developments, “do not talk about your family’s repression,” but also survival messages such as “study well” and “choose a safe profession.” An example reported by an 85 female Georgian survivor of family persecution (1):

“I remember my daddy… his face was as pale as the shirt. He went down on his knees before my mother, embraced her legs and told her, ‘wife, please, do everything to provide education to these kids’ … my mother swore before him, saying she would do it, even if she had to sacrifice her own life” (1).

In this Georgian study, there was a strikingly high number of doctors among the members of the second generation, as this profession was considered relatively safe, even under conditions of deportations and prison camps and left alive for the use of their professional skills. The study brings examples of people who originally wanted to study other disciplines but were successfully persuaded by their parents to study medicine for the reasons mentioned (up to the third generation).

A further study collected survival messages from Russians in their homeland and the US diaspora in the second and third follow-up generations (40). It particularly focused on survival messages related to coping with fears and general attitudes, e.g., ‘Do not be afraid’, ‘Do not show your fears and pain!’, ‘Do not trust!’ or a variety of others like *“It is good to limit yourself, stock up, save resources, do not plan anything in advance, take your time-consider carefully’, ‘Be like everyone else. Do not attract attention. … You must own nothing that will hold you back… Do not ask for help and do not beg for mercy!, You must be independent so you do not have to rely on people whom you cannot trust*” (21). These findings may have a validity limitation, as they do not distinguish with certainty between outcomes of the Stalinist/post-Stalinist repression and Second World War experiences. For future studies of survival messages, it is certainly important to achieve a clear separation of the respective origin of the phenomena, despite the methodological difficulties.

In a comparative study between Ukraine (Stalinist repression) and Israel (Holocaust) around 2015 researchers found different typical contents of messages. Common in Ukraine were messages of the coping-related type ‘Keeping a low profile’ in order not to become a victim again in case of new political developments. In Israel the communication-related type ‘Do not talk about your family’s repression’ prevailed, which was interpreted as a general ascetic-modest attitude towards life (41).

In summary, an astonishing number of studies (see Supplementary Table S1) show the outlines of a pattern of individual and family consequences of the Stalinist repression. With the many studies on intergenerational survival messages HT-related research on Stalinist and post-Stalinist repression can provide a successful example of unique, interesting research opportunities, which can offer an important starting point for psychological treatments, explaining possible family dysfunctions.

### Generalized mistrust and political sentiments

HT effects also include social pathologies or collective enmity attitudes. Empirical evidence for this is available from recent behavioral economics and political science studies that focus on mistrust and long-lasting political enmities. This type of studies correlates HT with opinion polls from several hundreds to thousands of people living in Eastern Europe today. Amongst those poll data, generalized trust in others is an essential parameter because it is a basis indicator for various political or economic exchange processes and that underlie socioeconomic prosperity of a society (42).

In the most extensive study, with data collected in 2016 by a World Bank study in Russia, living in geographic proximity to former Stalinist Gulag prison camps was associated with current lower social trust and civic engagement (43). Using a very careful methodology, this large-scale study controlled for a dozen of alternative variables, but they did not diminish this association. In an analog East German study, the numerical density of former Stasi security police informal networks (in the 1980s) in geographic areas was associated the trust scores in the same areas from the 2010s and likewise confirmed (44). Another Russian study (before the 2014 invasion of Crimea and eastern Ukraine), examining political enmities in the form of anti-Putin voting behaviors, showed that this was also positively associated with proximity to Gulag camps (45).

Two social science studies from Ukraine used anti-Soviet or anti-Russian political attitudes as indicators of long-term effects of Stalinist repression. The first study showed that the strongest after-effects were in the geographical areas with the highest mortality rates from the repression-induced Holodomor. Where death rates were particularly high (30% starvation deaths south of Kiev near Poltava and Tetiyiv), there was a pronounced anti-Soviet or anti-Russian attitude even after more than 70 years, which was not the case in the areas with the lowest Holodomor death rates (death rate of 1–2% in Donetsk region). In the second study, Crimean Tatars descendants (an ethnic group deported to Siberia under Stalin) were also examined for long-term effects, among others political enmities, and found to have the same kind of effects (46).

These studies on what can be called the social psyche impressively complement the pattern of long-term consequences of historical trauma. The lack of generalized trust and sometimes own societal commitment can be still found in many Eastern and Central European countries. The aforementioned ‘post-communist syndrome’ (3), consisting of learned helplessness, incivility, and lack of civic virtues, can be seen as possible consequence of these processes. However, it is likely that Russia’s new military aggression will interact with some of these psychosocial processes, whether in a specific strengthening or weakening of such sentiments.

## Prospects for remedy interventions and social healing

From the beginning, the historical trauma concept included as a genuine component fresh reflection on remedies and healing, including forms of commemoration, identity reflection or strengthening and psychosocial interventions (6). However, in most of these areas such specific approaches still seem to be lacking for victims and subsequent generations of Stalinism and post-Stalinism repression. The victims’ or commemorative organizations complain about this repeatedly. Some countries have used lustration procedures to exclude former perpetrators, especially of the respective secret police, from further holding political responsibilities. Memorials and tributes were established at different speeds - quickly in the Baltic countries, very hesitantly in most other countries, including (East) Germany. For instance, in Berlin there had already been the very well-visited Stasi prison museum Hohenschönhausen since 1992 but it was not until December 2022 that a central memorial for the victims of Stalinism was politically decided. In order to maintain domestic political peace in the countries during the transition period, the interests of the victims and their descendants were usually put on the backburner.

Unlike in other regions of the world with historical trauma, there were no ‘truth and reconciliation commissions’ in which victims and perpetrators interacted directly. A principle of these commissions was that victims could speak out about their politically inflicted suffering and that former perpetrators would face up to their responsibility. Also, the model that Israeli-German psychotherapists had conducted similar therapeutic groups for Holocaust victim and perpetrator descendants (47) was not extended to Stalinism victims and perpetrators. There are few case reports from centers for psychological therapy that had profiled Stalinism and post-Stalinism victims (4, 24, 25).

In the absence of direct evidence on remedies, the paper concludes with a broader look at international attempts to overcome the consequences of HT. A study on Israeli Auschwitz memorials visits suggests lasting effects on both the descendants of those affected and potentially the majoritarian society. In a longitudinal control group design, from a large number of high school students, half of them visited the Holocaust memorial Auschwitz and half of whom did not (48). The two groups differed in terms of emotional-cognitive effects attributable to the memorial. Strongest was the effect of the visiting group on identification with the victims, followed by an increase in empathy for other victim groups and, to a lesser extent pride in the ancestors - and this despite the fact that hopelessness and fear also increased slightly. This encouraging finding provides a strong argument that memorial visits can promote prosocial attitudes and thus contribute to social reconciliation. In fact, this is a demonstration of an interdisciplinary approach to research that should continue to pursue the HT topic in the future.

Also, countries in further-flung regions of the world with historical trauma also have something to offer. In Rwanda, socio-therapeutic interventions were carried out on a large scale after the genocide against the Tutsis in 1994. For example, groups of around a dozen people, consisting of former victims, perpetrators, and bystanders, met under the guidance of trained facilitators to discuss topics such as safety, trust, care, respect, rulemaking, careful handling of memories. There was no direct victim-perpetrator confrontation or discussion of the atrocities experienced by the victims. These groups, which lasted at least 15 weeks, were positively evaluated in a control group design - and further evaluations of them continue to this day (49, 50).

Although, as indicated in the methodological limitations section, previous research gaps are apparent, the density of evidences indicates that the HT concepts have both explanatory as well as healing potential. The most important message builds up around the fact that there are individual and societal consequences of long-ago collective trauma. Using the suggestions for those affected by the Eastern European historical consequences of political repression, the HT concept still provides useful new support for the problems of people who have so far remained largely in the shadows of public consciousness as well as public health attention.

## Data availability statement

The original contributions presented in the study are included in the article/Supplementary material, further inquiries can be directed to the corresponding author.

## Author contributions

The author confirms being the sole contributor of this work and has approved it for publication.

## Conflict of interest

The author declares that the research was conducted in the absence of any commercial or financial relationships that could be construed as a potential conflict of interest.

## Publisher’s note

All claims expressed in this article are solely those of the authors and do not necessarily represent those of their affiliated organizations, or those of the publisher, the editors and the reviewers. Any product that may be evaluated in this article, or claim that may be made by its manufacturer, is not guaranteed or endorsed by the publisher.
