# Supplementary material for: How to deal with the past? How collective and historical trauma psychologically reverberates in Eastern Europe
Source: Front Psychiatry. 2023 Aug 24;14:1228785. doi: 10.3389/fpsyt.2023.1228785 (PMC10483133; doi:10.3389/fpsyt.2023.1228785)
Supplement: Supplementary file 1 [file Table_1.DOCX]

Supplement Table: Details of the papers or studies described in the text

|  | Authors (year) | Metho-dology | Sample (country) | Measures | Main Results | Comments |
| --- | --- | --- | --- | --- | --- | --- |
| Trauma concept and extend |  |  |  |  |  |  |
| Totalitarian trauma (TT) | Javakhishvili (2018) | interpret. | Sample cases (Georgia) | - | TT results in forced division into 3 groups: victims, perpetrators, bystanders |  |
|  | Cherepanov (2021) | interpret. | Extensional definition | - | TT function as abusive power and control with hard and soft power tactics against victims. Randomness of the terror: No one is safe. | Reference to Duluth Model (Domestic violence) |
| Contemporary margina-lisation and discrimina-tion |  |  |  |  |  |  |
| Pro Stalin attitudes | Levada Institute (2022) | quant. | N = 1600 representative Population (Russia) | Survey items | Approval of Stalin: 2008: 32% positive -> 2018: 56% positive |  |
| Indifference/rejection towards victims | Best & Hoffmann (2008) | quant. | N=1009 Representative populat. (Germany) | Survey items | Disinterest in dealing with the past: 19%.  Rejection of financial compensation for former political victims: 33% |  |
| Associated psychosocial processes |  |  |  |  |  |  |
| Manifestation in the victim generation | Bichescu et al (2005) | quant. | N = 57 former polit. prisoners (Romania) | Structured clinical interview | PTSD (lifetime) 54%  PTSD (current) 31% |  |
|  | Galiene & Kazlauskas (2005) | quant. | N = 774 former polit. prisoners (Lithuania) | Ad hoc self-report scale | Current symptoms: Flashbacks 57%, constantly feeling tense 23% | No PTSD diagnoses, but its core symptoms |
|  | Maercker & Schützwohl (1997) | quant. | N = 146 former polit. prisoners  (East-Germany) | Structured clinical interview | PTSD (lifetime) 60%  PTSD (current) 30% |  |
|  | Rutkowski et al (2015) | quant. | N = 262 former polit. prisoners incl. 1/3 Nazi terror victims (Poland) | DSM-IV symptom list | PTSD (lifetime) 71% |  |
|  | Solojed (2006) | qual. | N = 11 polit. persecuted persons  (Russia) | Ad hoc typical symptoms | Most typical symptoms and signs: fears, sleep disturbances, refusal of help, "survival strategies". |  |
|  | Bezo & Maggi (2015) | qual. | N = 15 Holodomor survivors (Ukraine) | Semi-structured interview, Content analysis | Common themes: Horror, fears, humiliation, shame | Investigated also 2^nd^ & 3^rd^ generation (see row below) |
|  | Zasiekina (2020)  Zasiekina & Zasiekin (2020) | quant. | N = 42 Holodomor survivors | Interview prompts, Linguistic markers | Text length predicted (+) by number of emotional words (fear, anger), moral judgement and time markers; (-) by cognitive processing markers (remember, know, recollect). | Confirmation of the previous findings on communication features of traumatised persons |
| Intergenerational transmission and survival messages | Bezo & Maggi (2015) | qual. | N = 30 2^nd^ & 3^rd^ generation Holo-domor survivors | Semi-structured interview, Content analysis | Common themes: Fear to take action, fear to change status quo, passivity, ‘slave mentality’ |  |
|  | Baker & Grippenreiter (1998) | quant. | N = 44 3^rd^ generat.(Russia) | Semi-structured interview with response categories | Two thirds were "cut off" from informa-tion about grandparents’ persecution, despite their close contact | Association with health, divorce rate, but not with living standard, finances |
|  | Böhm (2014) | quant. | N = 64 1^st^ and 2^nd^ generation (East Germany) | Self-report (Symptom check list) | Significantly higher than population sample: Mistrust, social withdrawal; correlation between 1^st^ and 2^nd^ generat.: mistrust r = .5, social withdrawal r = .3 | Evidence for lower social capital |
|  | Javakhishvili (2018) | qual. | N = 28 2^nd^ and 3^rd^ generation (Georgia) | Semi-structured interview, content analysis, e.g. transgenerational advices | Findings on intergenerational talk-  concealment dynamics, large number of  survival messages |  |
|  | Varga & Cherepanov (2017) | semiquan. | N = 58 1^st^ to 3^rd^ generation (Russia and diaspora) | Free enumeration of survival messages, thematic analysis | Content of the survival messages: traumatocentric,power sources, resources, survival skills |  |
|  | Zasiekina et al (2021) | qual. | N = 20 2^nd^ and 3^rd^ generation (Ukraine;  compar. w. Israel) | Semi-structured inter-views, e.g. messages to descendants | Basic messages: Food and nourishment/basic needs is the focus, meta-messages: remembering the past | Israeli Holocaust surviv. descendants: ascetic-modest attitudes |
| Distrust and political attitudes |  |  |  |  |  |  |
| Historically formed generalized distrust | Nikolova et al (2022) | quant. | 1500 data points in common (respon-dents and regions)  (Russia) | Life in transition Survey (2016)  Gulag camp location list 1950 (memorial.de) | Present-day trust association proximity to former Gulags  High proximity (<10 km): r = -.29  Low proximity (>50 km): r = -.06 | Interpreted as proof of historical impact after appr. 65 years |
|  | Lichter et al (2021) | quant. | 217 geographical units in common  (East-Germany) | Social econom. panel for trust factors (2015)  Density of Stasi spies in GDR counties (1980s) | Present-day reciprocal behavior to spy density, controlled model: adj. R^2^ = .18 | Interpreted as strong historical impact after appr. 30 years |
|  | Zhukov & Talibova (2018) | quant. | 165 geographical units in common  (Russia) | Election turnout 2003-2012; Memorial  Terror victims’ archive  (memo.ru) | With exception of election 2011: association strength: tau = -.01 to -.12 | Weak effect: more historical repression, less election turnout |
| Anti-soviet polit. Attitude etc. | Rozenas & Zhukov (2019) | quant. | 386 geographical units in common  (Ukraine) | Rayon level famine & ant-soviet etc. parameters | Famine – polit. attitude associations:  Anti-soviet partisans (1940s) Regress-Faktor = .35  Anti-soviet protest (1988-91) RF = .07  Anti-Russian votes (2002-14) RF = .19 | Weak to medium effects: higher Holodomor famine, more Anti-soviet actions |
|  | Lupu & Peisakhin (2017) | quant. | 300 pairs (family) 1^st^generation with 3^rd^generation Crimean Tartars | Repression dose (1^st^ g.); anti-Russian attit. & identity (3^rd^ g.) | Effects of 1^st^-generation repression on 3^rd^-generation attitudes:  Support of anti-Russian rebels r = .29  Support of own political leaders r = .21  Continued victimhood r = .21 | Zusatzanalysen (mit der 2. Generation) zeigten jeweilige intergenera-tional persistence of victimhood (r = .34-.47) |
